# Non-Canonical Roles of Apoptotic Caspases in the Nervous System

**DOI:** 10.3389/fcell.2022.840023

**Published:** 2022-02-23

**Authors:** Mahshid H. Dehkordi, Robert G. K. Munn, Howard O. Fearnhead

**Affiliations:** ^1^ Pharmacology and Therapeutics, National University of Ireland Galway, Galway, Ireland; ^2^ Department of Anatomy, University of Otago, Dunedin, New Zealand

**Keywords:** Apaf-1, dark, CED-4, differentiation, neuronal, axon, degeneration, plasticity

## Abstract

Caspases are a family of cysteine proteases that predominantly cleave their substrates after aspartic acid residues. Much of what we know of caspases emerged from investigation a highly conserved form of programmed cell death called apoptosis. This form of cell death is regulated by several caspases, including caspase-2, caspase-3, caspase-7, caspase-8 and caspase-9. However, these “killer” apoptotic caspases have emerged as versatile enzymes that play key roles in a wide range of non-apoptotic processes. Much of what we understand about these non-apoptotic roles is built on work investigating how “killer” caspases control a range of neuronal cell behaviors. This review will attempt to provide an up to date synopsis of these roles.

## Introduction

**FIGURE 1:**
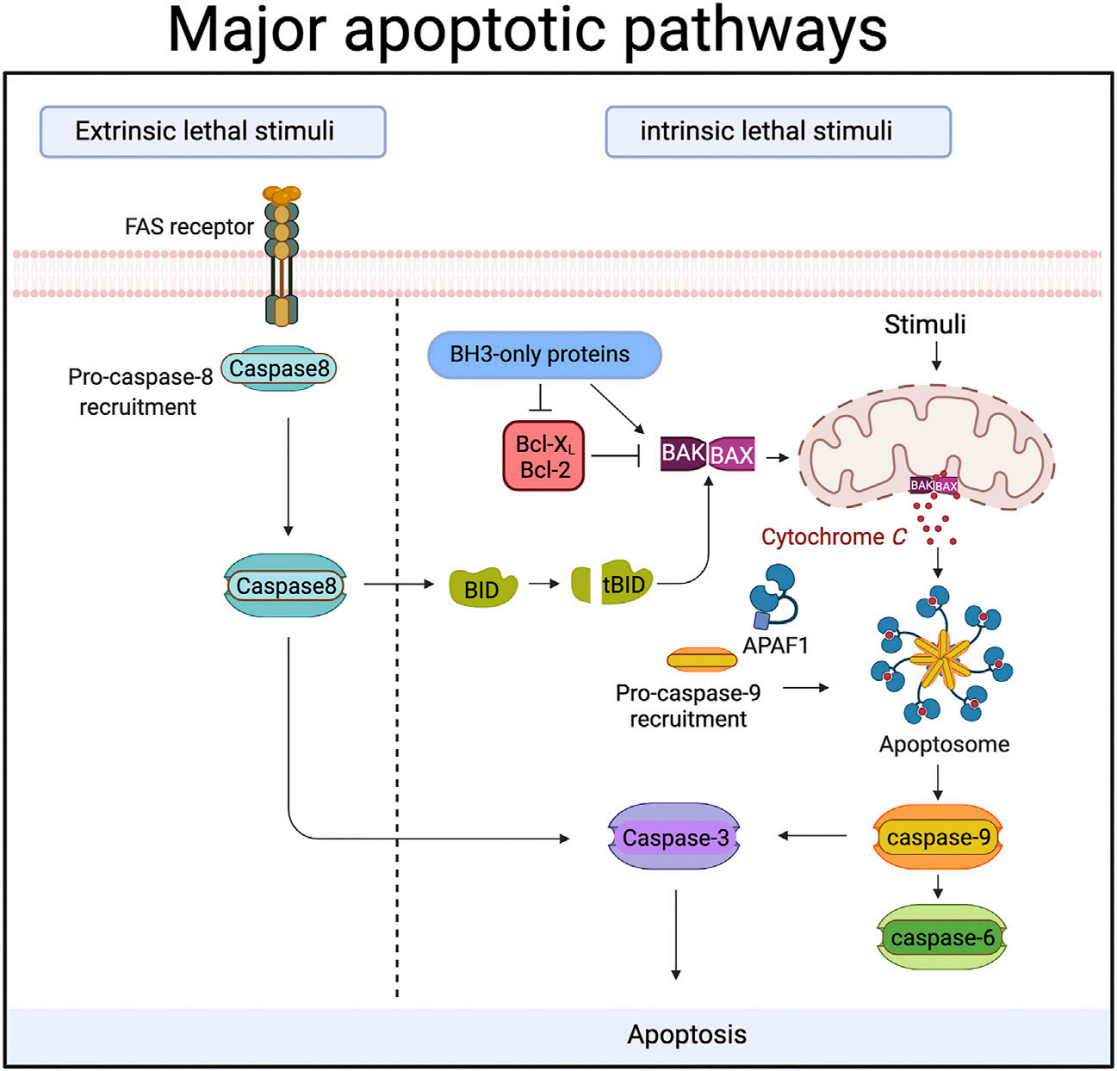
During apoptosis “executioner” caspases (caspase-3,-6 and -7) are activated by upstream “initiator” caspases (caspase-8 and caspase-9). In the extrinsic or death receptor pathway caspase-8 is activated by ligands binding to members of the death receptor family. Ligand binding triggers the recruitment of proteins to a large complex, the Death Inducing Signalling Complex (DISC). Caspase-8 is amongst those proteins, and its recruitment drives the autocatalytic processing and activation of caspase-8. In some cells caspase-8 then cleaves and activates caspase-3 directly. In other cells, caspase-8 cleaves a protein called Bid, an event that allows the cleaved Bid to activate the intrinsic or mitochondrial apoptotic pathway. In the mitochondrial death pathway signals cause the release of proteins from the inner mitochondrial membrane space. One of these proteins, cytochrome *c*, then binds to a cytosolic protein called Apaf-1. Cytochrome *c* binding causes Apaf-1 oligomerization and the formation of a protein complex (the apoptosome) that recruits and activates caspase-9. Caspase-9 is then able to activate the downstream effector caspases. Proteins of the Bcl-2 family control the release of cytochrome *c* from mitochondria, and can be both pro- and anti-apoptotic. Bax and Bak are pro-apoptotic members that sit in the mitochondrial membrane and are required for cytochrome c release. The pro-apoptotic activity of Bax and Bak is suppressed by the binding of anti-apoptotic members like Bcl-2 and Bcl-X_L_. BH3-only proteins like Bid, PUMA, Bim *etc* are pro-apoptotic Bcl-2 family members that act either by inhibiting anti-apoptotic Bcl-2 proteins, or by directly binding the pro-apoptotic proteins. Created with BioRender.com.

The process of apoptosis, a type of programmed cell death, is understood in some detail (Figure 1). Briefly, signals of various types that range from physiological to chemical insult activate pathways that culminate in the activation of a family of cysteine proteases called caspases. The cleavage of numerous proteins by these caspases brings about the death of the cell.

**FIGURE 2:**
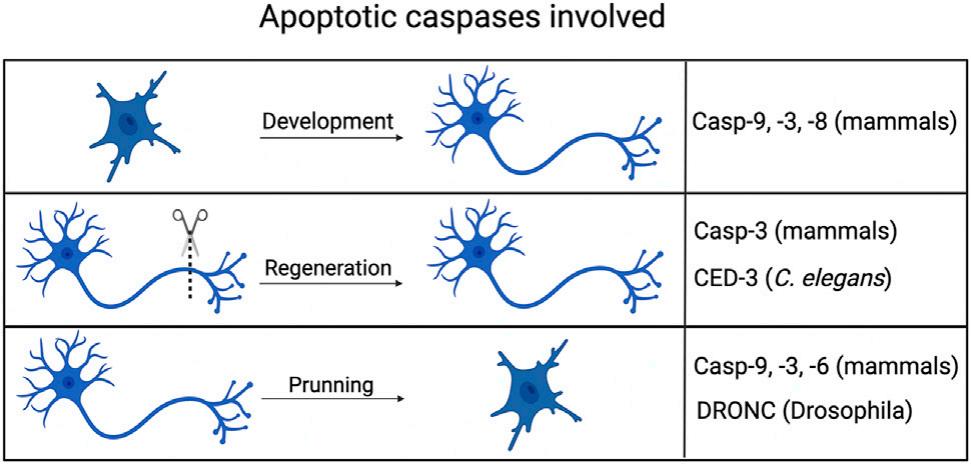
Non-apoptotic axonal processes involving apoptotic caspases. Caspases typically associated with cell death in mammals, flies and worms all play roles in aspects of axonal processes, including development (guidance), pruning/degeneration and regeneration. Created with BioRender.com.

The first caspase identified, caspase-1 plays a central role in inflammation, processing pro-interleukin-1β to its mature form and also inducing pyroptosis (Fink and Cookson, 2006), a form of necrotic cell death. Other caspases, notably caspase-4 and -5 also play pro-inflammatory and pyroptotic roles (J. Shi et al., 2014). However, it was the realization that apoptotic cell death in the nematode *Caenorhabditis elegans* relied on a caspase called CED-3 (Yuan, 1993) (Figure 2) that led to the discovery of mammalian apoptotic caspases and the elucidation of the molecular pathways that cause cell death (Figure 1). Much subsequent research identified the roles of caspases-2, 3, -7, -8 and -9 in cell death, demonstrating the ability of “initiator caspases” like caspase-2, -8 and -9 to couple upstream signals to the activation of “effector caspases”, like caspase-3 and -7. Caspase-6 is an apoptotic effector caspase and is typically placed downstream of caspase-3 (Slee et al., 1999). During apoptosis it brings about several changes in nuclear architecture (Ruchaud, 2002).

Caspase-dependent cell death is important in many contexts, playing key roles during tissue development and regeneration, regulating cell numbers and providing a mechanism for removing infectious or cancerous cells. However, the caspases that kill cells are far more versatile than we first appreciated. Roles for caspases typically associated with cell killing were identified in several types of cell differentiation, including the differentiation of neurons. These discoveries fundamentally altered how “killer” caspases were viewed. Where previously caspase activation was seen as synonymous with cell death, it became clear that activation of caspases is not necessarily a cellular death sentence and that activated killer caspases can induce other cell fates/behaviors. Exactly how cell death is avoided, and how killer caspases induce non-cell death fates is an area of active research, much of it conducted using neuronal models. This review is an attempt to integrate what we know about the non-canonical activity of apoptotic pathways in the nervous system, describing the relevant processes and where possible the signals that activate caspases, the regulation of caspase activity that prevents cell death and the substrates cleaved during non-apoptotic processes.

## Non-Apoptotic Roles of Caspases in Axonal Growth and Routing During Development and After Injury

During development neurons extend axons along chemotactic gradients to establish a network of interactions with other neurons. Apoptotic caspases regulate many aspects of axonal behavior (Figure 2). Axonal growth depends upon the growth cone at the tip of an axon, a specialized structure with a high dynamic cytoskeleton that responds to cues that guide an axon to proper target. Genetic and pharmacological experiments have clearly established that apoptotic caspases play important roles in this process. *In vitro* inhibition of caspase-3 activity also reduced the extension of neurites from neurosphere bodies, a process which mimics the dendritic and axonal branching that occurs *in vivo* (Fernando et al., 2005). The neural cell adhesion molecule (NCAM) is a cell-cell adhesion protein and is required for proper axonal guidance (Walsh and Doherty, 1997) and NCAM^−/-^ mice show significant defects in axonal growth and pathfinding (Cremer et al., 1997). Caspase-3 and caspase-8 inhibitors block NCAM-dependent neurite outgrowth in cultured mouse hippocampal neurons (Westphal et al., 2010). Ligand bound NCAM clusters in lipid rafts, triggering several downstream signaling pathways and promoting neurite outgrowth (Maness and Schachner, 2007). NCAM also binds to caspase-8.

### Activating Caspases During Axonal Growth and Routing

Initiator caspases, like caspase-8 can be activated by dimerization following induced proximity (Shi, 2004), so NCAM clustering may bring caspase-8 molecules together, causing dimerization and activation. Once active, caspase-8 can in turn activate caspase-3. It is proposed that caspase-3 then cleaves spectrin, altering the cytoskeleton and allowing neurite outgrowth. Increased caspase-3 is also seen in retinal neurons responding to the chemotrophic signals lysophosphatidic acid or netrin. Caspase inhibitors prevent this chemotrophic response (Campbell and Holt, 2003).

Caspase-3 and -9 activation has also been detected at the axonal branch points of retinal ganglion cells (Campbell and Okamoto, 2013). The role of caspase-9 in activating caspase-3 in the mitochondrial pathway in other contexts suggests that the mitochondrial pathway is important in this instance too. In support of this idea, axons are misrouted and synapse formation by sensory neurons is impaired in Apaf-1 null mice and caspase-9 null mice. The cleavage by caspase-9 of semaphorin 7A, a protein necessary for proper axonal growth appears to explain these deficits (Ohsawa et al., 2009, 2010). This is particularly interesting as there are relatively few caspase-9 substrates known compared to effector caspases like -3 and -7, and during apoptosis the role of caspase-9 appears to be primarily the activation of these effectors. Cleavage of semaphorin is a rare example of a non-lethal caspase substrate for caspase-9 whose cleavage is functionally important. Interestingly, NCAM and neuron-glia cell adhesion molecule (NgCAM), another protein prevalent on axons and a promoter of neurite growth and axonal fasciculation, are also caspase-3 substrates (Westphal et al., 2010; Weghorst et al., 2020), although the functional significance of this cleavage is not known.

### Caspase Substrates Cleaved During Axonal Growth and Routing

The cleavage by caspase-3 of cytoskeletal growth cone proteins (Campbell and Holt, 2003) as well as proteins like Gap43 that regulate growth cones (Denny, 2006) has also been demonstrated, leading to the suggestion that caspase-mediated remodelling of the cytoskeleton is central to axonal growth and guidance (reviewed by (Kellermeyer et al., 2018)). During apoptosis, caspase-3 cleaves actin, producing a 15 kDa fragment and causing condensation and fragmentation of the actin network (Mashima et al., 1997). Actin remodelling is also seen in the growth cones in the termini of developing neurons (Pacheco and Gallo, 2016). Interestingly, the 15 kDa fragment is detected in non-apoptotic neurons from the aged and from Alzheimer’s disease patients and co-localizes with an active form of caspase-3 (Rossiter et al., 2000), although the significance of this non-apoptotic caspase activation for the disease’s pathology remains uncertain. More recently, proteomic studies of proteins cleaved in the chick auditory brainstem have uncovered a non-apoptotic role for caspase-3 in cell-cell communication (Weghorst et al., 2020). The authors reported that many of the substrates cleaved were associated with non-apoptotic functions of caspases. Analysis of the substrates also revealed a disproportionately high level of proteins found in extracellular vesicles (EVs), a finding corroborated by proteomic analysis of EVs from the auditory brainstem. This analysis also identified NCAM and NgCAM in the EVs. These data suggest that caspase-3 activity may affect development of the auditory brainstem by modifying the cargo of EVs. Thus, despite many uncertainties, it appears that cleavage of proteins by caspase-3, -8 and -9 can regulate different cellular behaviors that are key for axon growth and guidance.

### Caspases in Axonal Regeneration

Axon growth and routing also occurs during the regeneration of broken axons. In mammals, caspase-3 inhibitors, but not a caspase-9 inhibitor block axon regeneration in dorsal root sensory neurons by preventing the formation of the growth cone (Verma et al., 2005). Inhibitors of caspase-3 or of calpain (a calcium-activated protease) also block axon regeneration in dorsal root ganglion cells (Öztürk et al., 2013). This process also involves local increases in protein translation at the site of injury (Verma et al., 2005), raising the possibility that a local increase of caspase activators is a key event. However, there are alternative explanations: after axotomy the local concentration of calcium rises into millimolar range (Ziv and Spira, 1995) before falling back to normal levels. These calcium fluxes are likely to activate the calcium sensitive protease, calpain. In apoptotic contexts calpain inhibitors or siRNA against µ-calpain block caspase-3 activation (Varghese et al., 2001), so perhaps calpain may play a role in growth cone formation and regeneration by activating caspase-3.

**FIGURE 3:**
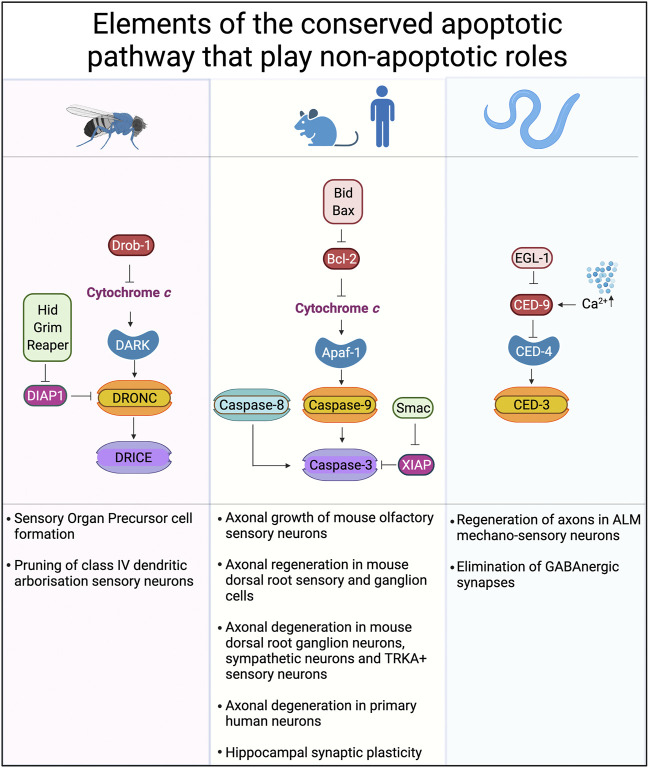
Conserved cell death pathways in mice, flies and worms that play non-apoptotic roles. The core proteins of the cell death machinery are highly conserved: Apaf-1, DARK and CED-4 play similar roles in caspase activation. Caspase-9, DRONC, and CED-3 are caspases activated directly by Apaf-1 or its homologs. Downstream of caspase-9 and DRONC are caspases-3, -6 and -7 in mammals while DRICE and DCP-1 are downstream of DRONC in *Drosophila*. Inhibitor of apoptosis proteins (IAPs), such as XIAP, DIAP-1 and others play key roles in binding and so inhibiting the activity of caspases. Created with BioRender.com.

In *C. elegans*, CED-4 (which is the nematode homolog of Apaf-1) and CED-3, (the homolog of caspase-9) are key proteins in axonal regeneration after axotomy of ALM mechano-sensory neurons (Pinan-Lucarre et al., 2012). The axonal regeneration is not dependent on EGL-1, the upstream regulator that controls CED-4 during apoptosis (Figure 3). Instead, CED-4 appears to be locally activated by signals arising from axonal damage and involves increased levels of intracellular calcium. These calcium fluxes activate the conserved Dual Leucine Zipper Kinase-1 (DLK-1) regeneration pathway to induce axonal regeneration (Hammarlund et al., 2009; Yan et al., 2009; Shin et al., 2012) upstream of CED-4/CED-3. As DLK-1 can induce local protein translation, it is possible that this is important for CED-3 activation. While DLK-1 is part of a conserved regeneration pathway in mammals, millimolar levels of calcium are known to bind and inhibit Apaf-1 (Bao et al., 2007) and Apaf-1 proteolysis is triggered by increases in calcium (Reimertz et al., 2001) so the role of Apaf-1 in mammalian axonal regeneration is not clear. Which proteins are cleaved by CED-3 during non-canonical processes is poorly understood, but cleavage of LIN-14, LIN-28 and DISL-2, which are key regulators of development involved in microRNA processing, is seen in non-apoptotic context (Weaver et al., 2014).

## Non-Apoptotic Roles of Caspases in Axon and Dendrite Degeneration During Development

While correct guidance of axons during development and recovery from insult is one possible function of non-apoptotic caspase cascades, so too is the remodeling of dendritic arbors that is critical for ongoing neuronal plasticity. Class IV dendritic arborisation (ddaC) sensory neurons are extensively remodeled during *Drosophila* metamorphosis, with old dendrites being pruned away from the cell body before new dendrites are extended to make new sets of contacts. The destruction of the dendrite arbor requires DRONC, the *Drosophila* homolog of caspase-9. During DRONC-dependent pruning of dorsal dendritic arborisation C neurons in *Drosophila*, DRONC activity is confined to the dendrites being pruned (Williams et al., 2006).

### Activating Caspases During Axon Pruning

During apoptosis in *Drosophila* DRONC is activated by the Apaf-1 homolog, DARK. Kang et al. (2017) reported DRONC-dependent axon pruning of ddaC neurons and that this occurred in DARK null flies, suggesting a DARK-independent activation mechanism. The basal level of DRONC activity is held in check by *Drosophila* homolog of X-linked inhibitor of apoptosis protein (XIAP), DIAP-1. DIAP-1 itself is antagonized by the pro-apoptotic proteins Grim, Reaper, Hid and Scythe (Silke and Meier, 2013). Basal levels of DRONC activation are known to play roles in *Drosophila* development being freed of DIAP-1 inhibition (Shinoda et al., 2019) and pruning also relies on the basal levels of activated DRONC. Consistent with this idea, the non-apoptotic remodelling of sensory neurons is dependent on the DIAP-1 inhibitors, Reaper, Hid and Grim (Mukherjee et al., 2021), and the tight control of Hid levels is vital for the process (Bhogal et al., 2016). The DARK-independent mechanism for sub-lethal activation of DRONC during pruning involves Tango7 (Kang et al., 2017), a protein that has also been linked to apoptosome regulation during spermatogenesis in *Drosophila* (D’Brot et al., 2013). Thus the DRONC activation occurs by distinct mechanisms depending on whether an apoptotic or non-apoptotic process is underway, the first involving an Apaf-1 homolog and the second critically dependent on inhibitor of apoptosis proteins.

Apaf-1-independent but caspase-dependent pruning also occurs in mammalian cells (Cusack et al., 2013), but how the caspases are activated is less well understood. In mammals, caspase-9 can be activated independently of Apaf-1 in a pathway triggered by the Patched receptor (Fombonne et al., 2012) or by the Deleted in Colorectal *Cancer* (DCC) dependence receptor (Forcet et al., 2001). There is also evidence that basal levels of active caspase-3 held in check by XIAP are freed to drive axon degeneration by XIAP degradation (Schoenmann et al., 2010; Ertürk et al., 2014). This is supported by several lines of evidence: XIAP regulates axonal degeneration (Unsain et al., 2013), and XIAP null mice have fewer synapses and learning and memory deficits (Gibon et al., 2016). These findings may be relevant to neurodevelopmental abnormalities: overexpression of E6AP is linked to autism and leads to significant loss of dendritic arborization. E6AP ubiquitinylates XIAP, increasing caspase activity and causing local degeneration and retraction at the tips of dendritic branches (Khatri et al., 2018). XIAP is also regulated by Smac/Diablo and Omi/Htr2A, two proteins that are released from mitochondria during apoptosis and that bind XIAP, relieving caspase inhibition (Ekert and Vaux, 2005). Although loss of Omi/Htr2A is neuroprotective (Martins et al., 2002), and Omi/Htr2A has been linked to Parkinson’s (Strauss et al., 2005) suggesting important roles in controlling neuron apoptosis, it is not clear whether non-apoptotic caspase activity involves the release of Omi/Htr2A (or Smac/Diablo). However, Omi/Hrt2A does play a non-apoptotic role as a serine protease in neurons, controlling the distribution of mitochondria along axons by cleaving vimentin (Lucotte et al., 2015). Caspase dysregulation has been implicated in Down Syndrome (DS), but has largely been linked to cell death through traditional apoptotic signaling. However, there are changes in the dendritic arbor of hippocampal pyramidal cells in the Ts65Dn mouse model of DS (Uguagliati et al., 2021) that suggest a non-apoptotic mechanism. The protein DYRK1A regulates caspase-9 in the developing retina (Laguna et al., 2008) and the gene is amplified in DS (Laguna et al., 2013). This constellation of observations suggests a caspase-mediated pathway may be central to abnormal connectivity seen in DS neurodevelopment.

### Caspase Cleavage of RUFY3 Is Required for Axonal Degeneration

During axonal degeneration of mouse TRKA + sensory neurons a neuronal protein called RUFY3 is cleaved by caspase-3. Deletion of RUFY3 protects the axon, even when caspase-3 is activated, suggesting that RUFY3 is the key substrate in caspase-3-mediated axonal degeneration. Phosphorylation of RUFY3 at Ser34 prevents axonal degeneration, so local dephosphorylation of RUFY3 may provide a mechanism to spatially limit axon degeneration (Hertz et al., 2019). RUFY3 is also known to limit axonal growth, and loss of RUFY3 results in multiple axons that are shorter (Mori et al., 2007; Wei et al., 2014). This may be explained mechanistically by RUFY3 forming complexes with Fascin, an actin-bundling protein, and Drebrin 1 (DBN1) (Wei et al., 2014). DBN1 can regulate actin too (G. Wang et al., 2015), but it also binds CXCR4, a chemokine that regulates neuronal migration (Shan et al., 2021). These data hint at multiple roles for RUFY3 in both axonal growth and axonal degeneration although caspase-3 cleavage of RUFY3 during axonal growth has not been reported.

### The Role of Caspase-6 in Axonal Degeneration

The primacy of caspase-3 in axonal degeneration is not clear: Nikolaev et al. (2009) showed that caspase-6 was present and activated in dorsal root ganglion axons after withdrawal of NGF in a Bax-dependent manner, and that this activation was required for axonal degeneration. The dependence on Bax suggests that caspase-6 was activated by the mitochondrial pathway (see Figure 1). Sympathetic neurons in caspase-6 null mice also show less axonal degeneration (Uribe et al., 2012). This role of caspase-6 may have disease relevance as caspase-6 has been linked to Alzheimer’s (Gervais et al., 1999; Guo et al., 2004; Albrecht et al., 2007) and to Huntington’s disease (Graham et al., 2006; Graham et al., 2010). Caspase-6 inhibitors reverse the cognitive impairment caused by caspase-6 overexpression and loss of caspase-6 reduces other disease aspects in a mouse model of Huntington’s (Wong et al., 2015; Pakavathkumar et al., 2017). Bax-dependent caspase-6 activity during axonal degeneration was also implicated in another study (Schoenmann et al., 2010), but a more complex picture was presented. On NGF withdrawal both caspase-3 and caspase-6 activity increased, but blocking caspase activity alone was insufficient to protect the axons. However combining caspase inhibition with NAD^+^ did protect the axons, suggesting a caspase-dependent pathway working in parallel with an NAD^+^ sensitive pathway. Because loss of Bax provided a more profound protection, it appears that it lies upstream of both the caspase-dependent and the NAD^+^ sensitive pathway.

The Bax-dependence of caspase-6 activity during axonal degeneration suggests that Apaf-1, caspase-9 and caspase-3 lie upstream of caspase-6. However, caspase-6 can also be activated by other pathways. For example, axonal degeneration in primary cultures of human neurons involves activation of neuronal NLRP1, which then forms an inflammasome to activate caspase-1, and caspase-1 cleaves and activates caspase-6 (Kaushal et al., 2015). However the crystal structure of caspase-6 suggests that unlike other effector caspases (-3 and -7), caspase-6 could also be activated by self-cleavage (Wang et al., 2010). This autocatalytic activation is controlled by caspase-6 phosphorylation, which locks caspase-6 in an inactive state (Cao et al., 2012).

The substrates that caspase-6 cleaves to cause axonal degeneration are not clear. Proteomic and other approaches have identified substrates cleaved during apoptosis, such as Lamin A (Ruchaud, 2002), or during neuronal degeneration such as CBP/p300 (Rouaux et al., 2003). Others have identified ∼24 substrates in neurons (Klaiman et al., 2008) and ∼47 substrates in other cell types (Cho et al., 2013). The identified substrates have roles in the cytoskeleton, signaling, chaperones, protein synthesis regulation, metabolism, proteolysis and membrane and lipid binding, but none are tied specifically to axonal degeneration.

### Bcl-2 Family Members and the Control of Caspase Activation During Non-Apoptotic Processes

In mammals at least, Apaf-1 is controlled by cytochrome *c* release from mitochondria, an event that is regulated by pro- and anti-apoptotic members of the Bcl-2 family. Axons in sensory and sympathetic neurons are protected from pruning in Bax null animals (Nikolaev et al., 2009) or by overexpression of the anti-Bcl-2 family member, Bcl-X_L_ (Vohra et al., 2010). While permeabilization of the outer mitochondrial membrane does more than simply release cytochrome *c*, the observations that Bcl-2 family members also play roles in axonal regeneration and guidance is consistent with a role for Apaf-1 and caspase-9. Similarly, axon pruning in mammals requires Bax (Nikolaev et al., 2009; Schoenmann et al., 2010; Simon et al., 2012), caspase-9 (Simon et al., 2012), and caspase-3 (Schoenmann et al., 2010), which suggests apoptosome involvement, even though a role for Apaf-1 was not tested directly in these studies. Axonal degeneration has also been indirectly linked to the mitochondrial pathway (Maor-Nof et al., 2016): the dual specificity phosphatase-16 (DUSP-16) is required for the preservation of sensory axons *in vivo*, and its loss accelerated axonal degeneration. DUSP-16 negatively regulates the pro-apoptotic BH3-only protein PUMA which causes axonal degeneration (Simon et al., 2016) and is required for the degeneration caused by loss of DUSP-16.

### Bcl-2 Proteins May Play Roles That Do Not Involve Caspases

However, the Bcl-2 proteins also have non-apoptotic roles that are separate from their ability to regulate caspases (reviewed in (Glab et al., 2020)), so concluding there is a role for the mitochondrial pathway in a non-apoptotic process simply on the basis it is regulated by Bcl-2 proteins is inappropriate. For example, Bad^−/−^ mice are resistant to acute seizures induced by either kainic acid or pentylenetetrazole (Giménez-Cassina et al., 2012) and a DGN-specific Bad knockout is sufficient to reduce seizure-like activity. Bad functions to control neuronal excitability by regulating cellular metabolism: Bad increases glucose metabolism and cellular ATP in response to phosphorylation on Ser155 (mouse Bad) or Serine 118 (human Bad). Phosphorylation on Ser155 also blocks Bad’s apoptotic activity (Virdee et al., 2000). In contrast, the pro-apoptotic effect of Bad is increased by phosphorylation on serine 128 (Konishi et al., 2002). The change in intracellular ATP regulates an ATP sensitive potassium channel, thus influencing neuronal excitability (Ramón Martínez-François et al., 2018). The reduced ATP levels affect the likelihood of K_ATP_ channel opening in neurons, decreasing the excitability of the neurons and reducing the risk of seizures (Giménez-Cassina et al., 2012).

The BH3-only members of the Bcl-2 proteins, most typically described as inducers of apoptosis, can also play non-apoptotic roles in a range of cellular contexts (Esposti et al., 2001; Danial et al., 2003, 2008; Sinha and Levine, 2008; Giménez-Cassina et al., 2012) including in the nervous system. For example, the reactive (and non-apoptotic) astrocytes that contribute to disease pathology in a mouse model of Amyotrophic Lateral Sclerosis (ALS) have increased expression of the BH3-only proteins, Bid, Hrk and Bnip3L. These data suggest that the BH3-only proteins are contributing to the development of ALS (Duval et al., 2018). There are also roles reported in non-mammalian models: in *C. elegans* EGL-1 expression increases in the URX pair of sensory neurons after changes in oxygen levels without killing the cells (Cohn et al., 2019).

## Non-Apoptotic Roles of Caspases in Synaptic Plasticity

**FIGURE 4:**
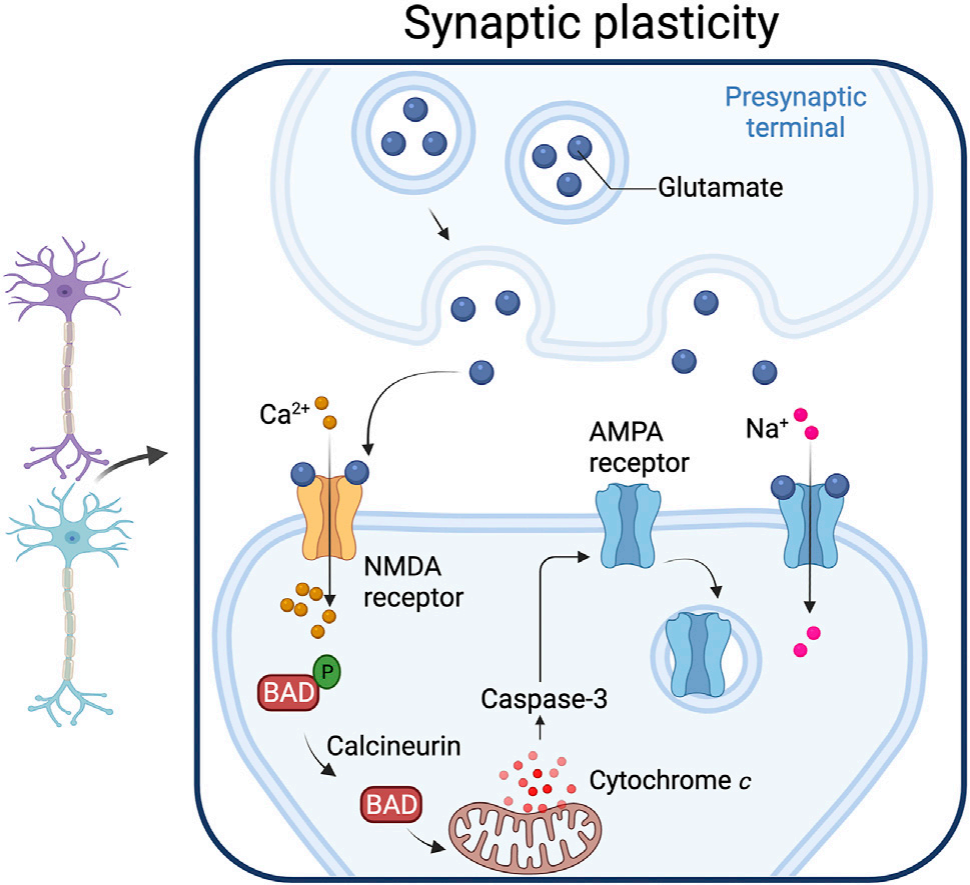
Synaptic plasticity. Caspase-3 activated by the mitochondrial pathway play a role in synaptic plasticity. Increased intracellular calcium levels activate calcineurin, a phosphatase that dephosphorylates the pro-apoptotic protein Bad, an event that leads to cytochrome *c* release from mitochondria. The resulting increase in caspase-3 produces a down-regulation of the AMPA receptor from the post-synaptic membrane. Adapted from “Long-Term Potentiation”, by BioRender.com (2021). Retrieved from https://app.biorender.com/biorender-templates.

Caspases bring about dynamic changes in axons and dendrites, but synapses are also dynamic structures. The long-term potentiation (LTP) or long-term depression (LTD) of synapses can involve many different discrete events, from phosphorylation or dephosphorylation of receptors, insertion or removal of receptors in the membrane, and signaling cascades that result in production of new proteins. Synaptic plasticity has been reckoned by many to underpin learning and memory as blockade of the mechanisms of LTP and LTD results in spatial memory deficits (D’Hooge and de Deyn, 2001). Pharmacological experiments have shown that caspase inhibitors also impair spatial memory formation (Dash et al., 2000), avoidance behaviour (Stepanichev et al., 2005), and auditory memory formation in birds (Huesmann and Clayton, 2006). Moreover, active caspase-3 is present in post-synaptic structures. Caspase inhibitors disrupt LTP in rat hippocampal neurons (Gulyaeva et al., 2003) suggesting that caspase activity plays important roles in controlling synaptic plasticity. Gene knockout studies substantiate this idea: caspase-3 null mice show impaired LTD (Li et al., 2010; Lo et al., 2015). Caspase-3-dependent LTD is accompanied by cytochrome *c* release, and mice lacking the pro-apoptotic Bcl-2 proteins, Bax and Bad also show a defect in LTD (Jiao and Li, 2011). These data strongly suggest that the mitochondrial pathway and Apaf-1 is playing a non-apoptotic role in LTD. Consistent with this idea, the elimination of GABAnergic synapses in *C. elegans* requires the Apaf-1 homolog CED-4 (Miller-Fleming et al., 2016). This collection of findings suggests that caspase cascades are intimately involved in ongoing plasticity in the mammalian brain, and may in fact have major molecular roles quite apart from the cell death mechanism with which they have been associated (Figure 4).

Whether minority MOMP plays a role in generating sub-lethal caspase activation during LTD is not known, and a role for Apaf-1 in LTD has not been demonstrated. Nonetheless, caspase-3 activity is required for the removal of AMPA receptors from the post-synaptic membrane and suppression of synaptic transmission. XIAP is also important in LTD (Gibon et al., 2016), and it appears to be regulated by FAIM-L (Moubarak et al., 2013), a protein that stabilizes XIAP and that is important for controlling LTD and axonal degeneration (Martínez-Mármol et al., 2016). SIVA-1 is a pro-apoptotic protein that plays a non-apoptotic role in LTD by modulating AMPAR internalization. SIVA-1 binds FAIM-L and disrupts the FAIM-L–XIAP interaction, increasing XIAP ubiquitination and caspase-3 activity (Coccia et al., 2020). How caspase activity brings about LTD is an area of active research. A proteomic approach identified neuronal caspase-3 substrates, including Gap43, which is required for AMPA receptor internalization, and showed that Gap43 is cleaved in post synaptic structures during LTD (Han et al., 2013). Another proteomic study identified caspase substrates in mouse synaptosomes (Victor et al., 2018), including known caspase substrates and novel substrates (like the proton pump subunit ATP6V1B2 and the N-ethylmaleimide-sensitive fusion protein). More recently a role for Bax, Bad and caspase-3 in controlling synaptic vesicle pools via autophagy has been proposed (Gu et al., 2021). These findings may be relevant for the treatment of neuropathic pain: peripheral nerve injury causes a downregulation of caspase-3 which prevents LTD and causes hypersensitivity to pain (Y. J. Wang et al., 2020).

### Non-Apoptotic Roles of Caspases in Neural and Neuronal Differentiation

In the fly, the sensory organ precursor cell (SOP) gives rise to mechano-sensory organs in the peripheral nervous system by dividing asymmetrically to make the shaft, socket, and sheath cells, and a neuron that comprise each sensory organ. The formation of SOPs is negatively regulated by DARK-dependent non-apoptotic caspase activation (Kanuka et al., 2005). Mechanistically, DRONC regulates this development by cleaving Shaggy, a negative regulator of Wingless signalling, which is required for SOP formation (Kanuka et al., 2005). DRONC activity is controlled by IKK-related kinase (DmIKKε)-mediated phosphorylation of DIAP-1. DIAP-1 phosphorylation increases DIAP-1 ubiquitinylation, which leads to a decrease in DIAP-1 levels through proteasome-mediated degradation (Kuranaga et al., 2006).

Caspase-3 is activated in non-apoptotic cells during mammalian neuronal differentiation in a cultured neurosphere model, and preventing this activation disrupts differentiation (Fernando et al., 2005). There is also evidence for the mitochondrial pathway playing a role during neuronal cell differentiation an siRNA against caspase-9 reduced neuronal differentiation in a cell culture model (Pistritto et al., 2012). Changes in calcium levels during the differentiation of neuronal cells have also been implicated in caspase activation (Rebellato et al., 2019). The mechanism of caspase activation is not clear, but it involves the low-voltage-dependent T-type calcium (Ca^2+^) channel Cav3.2 which mediates increased levels of Ca^2+^ that are required to activate caspase-3-dependent neurogenesis during cortical development (Rebellato et al., 2019).

## Controlling Apoptotic Caspases During Non-Apoptotic Processes

When considering the non-apoptotic roles of caspases there is an obvious question: why don’t the cells die? Caspases are cleaving substrates to alter cell behaviour, so there is expected to be a difference in identities of the proteins cleaved when different cell fates are induced and perhaps also differences in the level of cleavage. There are data consistent with these expectations, showing that substrate cleavage may be controlled during non-apoptotic processes by limiting both the amount and the sub-cellular localization of caspase activity.

### Controlling Levels of Caspase Activity

In *Drosophila* salivary cells undergoing remodelling there is a 30-fold increase in Reaper expression, compared to a 1000-fold increase in dying cells (Kang et al., 2017). The implication is that there is two orders of magnitude more caspase activity in dying cells, and that the decision between a non-apoptotic fate and an apoptotic fate is made at the level of Reaper expression. Low level caspase activity is involved in the emergence of neural precursor cells in *Drosophila* (Kanuka et al., 2005; Kuranaga et al., 2006). In mammalian cells control of XIAP and expression of Apaf-1 are reported to be important in determining the choice between death and differentiation (Wright et al., 2004; Potts et al., 2005; Johnson et al., 2007; Yin et al., 2007; Smith et al., 2009). At least one mechanism for controlling XIAP in non-apototic processes has been described (see above, (Moubarak et al., 2013; Coccia et al., 2020)), but in neurons it appears that the level of Apaf-1 limits the caspase activity (Wright et al., 2004).

How levels of Apaf-1 are controlled during differentiation is unresolved. In cancer cells Apaf-1 transcription is controlled by E2F-1 (Furukawa et al., 2002) and by TP53 (Rozenfeld-Granot et al., 2002), so the decrease in E2F-1 that accompanies exit from the cell cycle and that precedes differentiation may provide a straight forward explanation for a decrease in Apaf-1 that limits caspase activation during differentiation. Apaf-1 levels are also decreased in some cancers by microRNAs. Notably one miRNA that targets Apaf-1, miR-221, is increased during neuronal differentiation (Hamada et al., 2012) and during myogenesis (Liu et al., 2018), both systems where a decrease in Apaf-1 level appears to be important step during differentiation (Fernando et al., 2005; Dehkordi et al., 2020). Other microRNAs are reported to regulate Apaf-1 in cancers, but their roles in neurogenesis and myogenesis are not well described.

### Controlling Localization of Active Caspases

There is evidence that caspase localization is important in apoptosis. For example, in lung cancer cells the nuclear localization of active caspase-3 is linked to sensitivity to cancer chemotherapy (Joseph et al., 2001). During spermatid individualization caspases are activated by DARK to remodel the spermatid without apoptosis (Arama et al., 2003; Arama et al., 2007; Huh et al., 2004). This activity is localized through the interplay of an E3-ligase inhibitor, Soti and the IAP-like dBruce protein (Kaplan et al., 2010). In a different *Drosophila* model, Amcheslavsky et al. (2018) used cells lacking Drice (which are unable to undergo apoptosis) to show that Myo1D localizes DRONC to the plasma membrane where DRONC increases expression of NADPH-oxidase Duox, generating eROS that drives apoptosis induced cell proliferation. In DRONC-dependent salivary cell remodeling, Tango7 is required for the cortical localization of active DRONC (Kang et al., 2017). These data are very interesting as they show the activation of caspases in distinct sub-cellular domains being driven by different caspase-activation pathways. In *Drosophila* neurons caspase activity is restricted to dendrites during pruning, but is not seen in the soma or axon (Kuo et al., 2006; Williams et al., 2006). Nuclear localization of active caspase-3 is seen in non-apoptotic Bergmann Glial cells (Noyan-Ashraf et al., 2005). This activity appears to be important for the differentiation of these cells (Oomman et al., 2006).

## Future Perspectives

The diverse roles of caspases enumerated here reflect specific caspase-mediated cleavage events driving distinct non-apoptotic processes. This implies a clear delineation between the particular substrates that are cleaved during the different non-apoptotic processes. This perhaps most important in the axon, where both growth, regeneration and degeneration are produced by caspase activity. Part of the reason that caspases drive different outcomes lies in the caspases involved in each process. For example, caspase-3 appears to be important in growth, while caspase-6 appears key in degeneration. These two caspases have distinct sets of substrates (Cho et al., 2013; Julien et al., 2016), so this provides an immediate explanation for how two different outcomes are produced, even though we don’t know much about the particular proteins that are being cleaved. Different caspase-dependent outcomes occurring in different locations (*e.g.* the soma for differentiation or synapses during LTD) but induced by the same caspase are a different case, but may be explained by subcellular localization of the caspase activity or the relevant substrates, and there is evidence of this for caspase-3 as discussed above. Detailed proteomic analysis of the substrates involved in some of these neuronal processes has begun (Klaiman et al., 2008; Hertz et al., 2019), but a more detailed knowledge of how substrate cleavage produces different outcomes will improve our understanding of normal and diseased neuronal tissue as well as helping us understand how caspases kill in other cell types.

A related, and unresolved set of questions is how the caspases are activated. There is evidence for the mitochondrial apoptotic pathway being central in many of the non-apoptotic processes, but there are also less well-understood mechanisms involving dependence receptors, NCAM, and changes in intracellular calcium concentrations. These activation pathways seem likely to be linked to several different neurodegenerative diseases, and a detailed molecular understanding of the steps leading up to caspase activation may uncover promising new therapeutic targets.

## References

[B1] Albrecht S. Bourdeau M. Bennett D. Mufson E. J. Bhattacharjee M. LeBlanc A. C. (2007). Activation of Caspase-6 in Aging and Mild Cognitive Impairment. Am. J. Pathol. 170 (4), 1200–1209. 10.2353/ajpath.2007.060974 17392160 PMC1829454

[B2] Amcheslavsky A. Wang S. Fogarty C. E. Lindblad J. L. Fan Y. Bergmann A. (2018). Plasma Membrane Localization of Apoptotic Caspases for Non-apoptotic Functions. Dev. Cel 45 (4), 450–464.e3. 10.1016/j.devcel.2018.04.020 PMC5972739 29787709

[B3] Arama E. Agapite J. Steller H. (2003). Caspase Activity and a Specific Cytochrome C Are Required for Sperm Differentiation in Drosophila. Dev. Cel 4, 687–697. 10.1016/s1534-5807(03)00120-5 12737804

[B4] Arama E. Bader M. Rieckhof G. E. Steller H. (2007). A Ubiquitin Ligase Complex Regulates Caspase Activation during Sperm Differentiation in Drosophila. Plos Biol. 5 (10), e251–2287. 10.1371/journal.pbio.0050251 17880263 PMC1976628

[B5] Bao Q. Lu W. Rabinowitz J. D. Shi Y. (2007). Calcium Blocks Formation of Apoptosome by Preventing Nucleotide Exchange in Apaf-1. Mol. Cel 25 (2), 181–192. 10.1016/j.molcel.2006.12.013 17244527

[B6] Bhogal B. Plaza-Jennings A. Gavis E. R. (2016). Nanos-mediated Repression of Hid Protects Larval Sensory Neurons after a Switch in Sensitivity to Apoptotic Signals. Development (Cambridge) 143 (12), 2147–2159. 10.1242/dev.132415 PMC4920170 27256879

[B7] Campbell D. S. Holt C. E. (2003). Apoptotic Pathway and MAPKs Differentially Regulate Chemotropic Responses of Retinal Growth Cones. Neuron 37, 939–952. 10.1016/s0896-6273(03)00158-2 12670423

[B8] Campbell D. S. Okamoto H. (2013). Local Caspase Activation Interacts with Slit-Robo Signaling to Restrict Axonal Arborization. J. Cel Biol. 203 (4), 657–672. 10.1083/jcb.201303072 PMC3840933 24385488

[B9] Cao Q. Wang X.-J. Liu C.-W. Liu D.-F. Li L.-F. Gao Y.-Q. (2012). Inhibitory Mechanism of Caspase-6 Phosphorylation Revealed by crystal Structures, Molecular Dynamics Simulations, and Biochemical Assays. J. Biol. Chem. 287 (19), 15371–15379. 10.1074/jbc.M112.351213 22433863 PMC3346075

[B10] Cho J. H. Lee P. Y. Son W.-C. Chi S.-W. Park B. C. Kim J.-H. (2013). Identification of the Novel Substrates for Caspase-6 in Apoptosis Using Proteomic Approaches. BMB Rep. 46 (12), 588–593. 10.5483/BMBRep.2013.46.12.081 24195789 PMC4133863

[B11] Coccia E. Planells-Ferrer L. Badillos-Rodríguez R. Pascual M. Segura M. F. Fernández-Hernández R. (2020). SIVA-1 Regulates Apoptosis and Synaptic Function by Modulating XIAP Interaction with the Death Receptor Antagonist FAIM-L. Cell Death Dis 11 (2). 10.1038/s41419-020-2282-x PMC6997380 32015347

[B12] Cohn J. Dwivedi V. Valperga G. Zarate N. de Bono M. Horvitz H. R. (2019). Activity-Dependent Regulation of the Proapoptotic BH3-Only Gene Egl-1 in a Living Neuron Pair in *Caenorhabditis elegans* . G3: Genes, Genomes, Genet. 9 (11), 3703–3714. 10.1534/g3.119.400654 PMC6829140 31519744

[B13] Cremer H. Chazal G. Goridis C. Represa A. (1997). NCAM Is Essential for Axonal Growth and Fasciculation in the Hippocampus. Mol. Cell Neurosci. 8, 323–335. 10.1006/mcne.1996.0588 9073395

[B14] Cusack C. L. Swahari V. Hampton Henley W. Michael Ramsey J. Deshmukh M. (2013). Distinct Pathways Mediate Axon Degeneration during Apoptosis and Axon-specific Pruning. Nat. Commun. 4. 10.1038/ncomms2910 PMC4183061 23695670

[B15] Danial N. N. Gramm C. F. Scorrano L. Zhang C.-Y. Krauss S. Ranger A. M. (2003). BAD and Glucokinase Reside in a Mitochondrial Complex that Integrates Glycolysis and Apoptosis. Nature 424, 952–956. 10.1038/nature01825 12931191

[B16] Danial N. N. Walensky L. D. Zhang C.-Y. Choi C. S. Fisher J. K. Molina A. J. A. (2008). Dual Role of Proapoptotic BAD in Insulin Secretion and Beta Cell Survival. Nat. Med. 14 (2), 144–153. 10.1038/nm1717 18223655 PMC3918232

[B17] Dash P. K. Blum S. Moore A. N. (2000). Caspase Activity Plays an Essential Role in Long-Term Memory. Neuroreport 11, 2811–2816. 10.1097/00001756-200008210-00040 10976968

[B18] D’Brot A. Chen P. Vaishnav M. Yuan S. Akey C. W. Abrams J. M. (2013). Tango7 Directs Cellular Remodeling by the Drosophila Apoptosome. Genes Dev. 27 (15), 1650–1655. 10.1101/gad.219287.113 23913920 PMC3744723

[B19] Dehkordi H. M. Tashakor A. O’Connell E. Fearnhead H. O. (2020). Apoptosome-dependent Myotube Formation Involves Activation of Caspase-3 in Differentiating Myoblasts. Cel Death Dis. 11 (5). 10.1038/s41419-020-2502-4 PMC7198528 32366831

[B20] Denny J. B. (2006). Molecular Mechanisms, Biological Actions, and Neuropharmacology of the Growth-Associated Protein GAP-43. Curr. Neuropharmacology 4, 293–304. 10.2174/157015906778520782 PMC2475799 18654638

[B21] D’Hooge R. de Deyn P. P. (2001). Applications of the Morris Water Maze in the Study of Learning and Memory. Brain Res. Rev. 36 (1), 60–90. 11516773 10.1016/s0165-0173(01)00067-4

[B22] Duval N. Sumner W. A. Andrianakos A. G. Gray J. J. Bouchard R. J. Wilkins H. M. (2018). The Bcl-2 Homology-3 Domain (BH3)-Only Proteins, Bid, DP5/Hrk, and BNip3L, Are Upregulated in Reactive Astrocytes of End-Stage Mutant SOD1 Mouse Spinal Cord. Front. Cell Neurosci. 12. 10.3389/fncel.2018.00015 PMC5797550 29440992

[B23] Ekert P. G. Vaux D. L. (2005). The Mitochondrial Death Squad: Hardened Killers or Innocent Bystanders? Curr. Opin. Cel Biol. 17 (6), 626–630. 10.1016/j.ceb.2005.09.001 16219456

[B24] Ertürk A. Wang Y. Sheng M. (2014). Local Pruning of Dendrites and Spines by Caspase-3-dependent and Proteasome-Limited Mechanisms. J. Neurosci. 34 (5), 1672–1688. 10.1523/JNEUROSCI.3121-13.2014 24478350 PMC6827581

[B25] Esposti M. D. Erler J. T. Hickman J. A. Dive C. (2001). Bid, a Widely Expressed Proapoptotic Protein of the Bcl-2 Family, Displays Lipid Transfer Activity. Mol. Cell Biol. 21 (21), 7268–7276. 10.1128/mcb.21.21.7268-7276.2001 11585909 PMC99901

[B26] Fernando P. Brunette S. Megeney L. A. (2005). Neural Stem Cell Differentiation Is Dependent upon Endogenous Caspase-3 Activity. FASEB J. 19 (12), 1671–1673. 10.1096/fj.04-2981fje 16103108

[B27] Fink S. L. Cookson B. T. (2006). Caspase-1-dependent Pore Formation during Pyroptosis Leads to Osmotic Lysis of Infected Host Macrophages. J. Immunol. 202 (7), 1913–1926. 10.1111/J.1462-5822.2006.00751.x 30885987

[B28] Fombonne J. Bissey P. A. Guix C. Sadoul R. Thibert C. Mehlen P. (2012). Patched Dependence Receptor Triggers Apoptosis through Ubiquitination of Caspase-9. Proc. Natl. Acad. Sci. United States America 109 (26), 10510–10515. 10.1073/pnas.1200094109 PMC3387056 22679284

[B29] Forcet C. Ye X. Granger L. ronique Corset V. Shin H. Bredesen D. E. (2001). The Dependence Receptor DCC (Deleted in Colorectal Cancer) Defines an Alternative Mechanism for Caspase Activation. Proc. Natl. Acad. USA 98 (6), 3416–3421. 10.1073/pnas.051378298 PMC30668 11248093

[B30] Furukawa Y. Nishimura N. Furukawa Y. Satoh M. Endo H. Iwase S. (2002). Apaf-1 Is a Mediator of E2F-1-Induced Apoptosis. J. Biol. Chem. 277 (42), 39760–39768. 10.1074/jbc.M200805200 12149244

[B31] Gervais F. G. Xu D. Robertson G. S. Vaillancourt J. P. Zhu Y. Huang J. (1999). Involvement of Caspases in Proteolytic Cleavageof Alzheimer’s Amyloid-bPrecursor Proteinand Amyloidogenic AbPeptide Formation. Cell 97, 395–406. 10.1016/s0092-8674(00)80748-5 10319819

[B32] Gibon J. Unsain N. Gamache K. Thomas R. A. de Leon A. Johnstone A. (2016). The X-Linked Inhibitor of Apoptosis Regulates Long-Term Depression and Learning Rate. FASEB J. 30 (9), 3083–3090. 10.1096/fj.201600384R 27189977

[B33] Giménez-Cassina A. Martínez-François J. R. Fisher J. K. Szlyk B. Polak K. Wiwczar J. (2012). BAD-dependent Regulation of Fuel Metabolism and KATP Channel Activity Confers Resistance to Epileptic Seizures. Neuron 74 (4), 719–730. 10.1016/j.neuron.2012.03.032 22632729 PMC3361694

[B34] Glab J. A. Cao Z. Puthalakath H. (2020). Bcl-2 Family Proteins, beyond the Veil. Int. Rev. Cel Mol. Biol. Vol 351, 1–22. 10.1016/bs.ircmb.2019.12.001 32247577

[B35] Graham R. K. Deng Y. Carroll J. Vaid K. Cowan C. Pouladi M. A. (2010). Cleavage at the 586 Amino Acid Caspase-6 Site in Mutant Huntingtin Influences Caspase-6 Activation *In Vivo* . J. Neurosci. 30 (45), 15019–15029. 10.1523/JNEUROSCI.2071-10.2010 21068307 PMC3074336

[B36] Graham R. K. Deng Y. Slow E. J. Haigh B. Bissada N. Lu G. (2006). Cleavage at the Caspase-6 Site Is Required for Neuronal Dysfunction and Degeneration Due to Mutant Huntingtin. Cell 125 (6), 1179–1191. 10.1016/j.cell.2006.04.026 16777606

[B37] Gu Q. Jiao S. Duan K. Wang Y. X. Petralia R. S. Li Z. (2021). The BAD-BAX-Caspase-3 cascade Modulates Synaptic Vesicle Pools via Autophagy. J. Neurosci. 41 (6), 1174–1190. 10.1523/JNEUROSCI.0969-20.2020 33303681 PMC7888220

[B38] Gulyaeva N. Kudryashov I. E. Kudryashova I. v. (2003). Caspase Activity Is Essential for Long-Term Potentiation. J. Neurosci. Res. 73, 853–864. 10.1002/jnr.10730 12949912

[B39] Guo H. Albrecht S. Bourdeau M. Petzke T. Bergeron C. Leblanc A. C. (2004). Active Caspase-6 and Caspase-6-Cleaved Tau in Neuropil Threads, Neuritic Plaques, and Neurofibrillary Tangles of Alzheimer’s Disease. In American Journal of Pathology (Vol. 165, Issue 2, pp. 523–531). 10.1016/S0002-9440(10)63317-2 PMC1618555 15277226

[B40] Hamada N. Fujita Y. Kojima T. Kitamoto A. Akao Y. Nozawa Y. (2012). MicroRNA Expression Profiling of NGF-Treated PC12 Cells Revealed a Critical Role for miR-221 in Neuronal Differentiation. Neurochem. Int. 60 (8), 743–750. 10.1016/j.neuint.2012.03.010 22465943

[B41] Hammarlund M. Nix P. Hauth L. Jorgensen E. M. Bastiani M. (2009). Axon Regeneration Requires a Conserved MAP Kinase Pathway. Science 323 (5915), 802–806. 10.1126/science.1165527 19164707 PMC2729122

[B42] Han M. H. Jiao S. Jia J. M. Chen Y. Chen C. Y. Gucek M. (2013). The Novel Caspase-3 Substrate gap43 Is Involved in Ampa Receptor Endocytosis and Long-Term Depression. Mol. Cell Proteomics 12 (12), 3719–3731. 10.1074/mcp.M113.030676 24023391 PMC3861719

[B43] Hertz N. T. Adams E. L. Weber R. A. Shen R. J. O’Rourke M. K. Simon D. J. (2019). Neuronally Enriched RUFY3 Is Required for Caspase-Mediated Axon Degeneration. Neuron 103 (3), 412–422.e4. 10.1016/j.neuron.2019.05.030 31221560 PMC8024238

[B44] Huesmann G. R. Clayton D. F. (2006). Dynamic Role of Postsynaptic Caspase-3 and BIRC4 in Zebra Finch Song-Response Habituation. Neuron 52 (6), 1061–1072. 10.1016/j.neuron.2006.10.033 17178408 PMC1847391

[B45] Huh J. R. Vernooy S. Y. Yu H. Yan N. Shi Y. Guo M. (2004). Multiple Apoptotic Caspase Cascades Are Required in Nonapoptotic Roles for Drosophila Spermatid Individualization. PLoS Biol. 2 (1). 10.1371/journal.pbio.0020015 PMC300883 14737191

[B46] Jiao S. Li Z. (2011). Nonapoptotic Function of BAD and BAX in Long-Term Depression of Synaptic Transmission. Neuron 70 (4), 758–772. 10.1016/j.neuron.2011.04.004 21609830 PMC3102234

[B47] Johnson C. E. Huang Y. Y. Parrish A. B. Smith M. I. Vaughn A. E. Zhang Q. (2007). Differential Apaf-1 Levels Allow Cytochrome C to Induce Apoptosis in Brain Tumors but Not in normal Neural Tissues. Proc. Natl. Acad. Sci. USA 104 (52), 20820–20825. 10.1073/pnas.0709101105 18093951 PMC2409225

[B48] Joseph B. Ekedahl J. Lewensohn R. Marchetti P. Formstecher P. Zhivotovsky B. (2001). Defective Caspase-3 Relocalization in Non-small Cell Lung Carcinoma. Oncogene 20, 2877–2888. 10.1038/sj.onc.1204402 11420700

[B49] Julien O. Zhuang M. Wiita A. P. O’Donoghue A. J. Knudsen G. M. Craik C. S. (2016). Quantitative MS-based Enzymology of Caspases Reveals Distinct Protein Substrate Specificities, Hierarchies, and Cellular Roles. Proc. Natl. Acad. Sci. United States America 113 (14), E2001–E2010. 10.1073/pnas.1524900113 PMC4833263 27006500

[B50] Kang Y. Neuman S. D. Bashirullah A. (2017). Tango7 Regulates Cortical Activity of Caspases during Reaper-Triggered Changes in Tissue Elasticity. Nat. Commun. 8 (1). 10.1038/s41467-017-00693-3 PMC5605750 28928435

[B51] Kanuka H. Kuranaga E. Takemoto K. Hiratou T. Okano H. Miura M. (2005). Drosophila Caspase Transduces Shaggy/GSK-3β Kinase Activity in Neural Precursor Development. EMBO J. 24 (21), 3793–3806. 10.1038/sj.emboj.7600822 16222340 PMC1276714

[B52] Kaplan Y. Gibbs-Bar L. Kalifa Y. Feinstein-Rotkopf Y. Arama E. (2010). Gradients of a Ubiquitin E3 Ligase Inhibitor and a Caspase Inhibitor Determine Differentiation or Death in Spermatids. Dev. Cel 19 (1), 160–173. 10.1016/j.devcel.2010.06.009 20643358

[B53] Kaushal V. Dye R. Pakavathkumar P. Foveau B. Flores J. Hyman B. (2015). Neuronal NLRP1 Inflammasome Activation of Caspase-1 Coordinately Regulates Inflammatory Interleukin-1-Beta Production and Axonal Degeneration-Associated Caspase-6 Activation. Cel Death Differ. 22 (10), 1676–1686. 10.1038/cdd.2015.16 PMC4563782 25744023

[B54] Kellermeyer R. Heydman L. M. Mastick G. S. Kidd T. (2018). The Role of Apoptotic Signaling in Axon Guidance. J. Dev. Biol. 6 (4). 10.3390/jdb6040024 PMC6316149 30340315

[B55] Khatri N. Gilbert J. P. Huo Y. Sharaflari R. Nee M. Qiao H. (2018). The Autism Protein Ube3A/E6AP Remodels Neuronal Dendritic Arborization via Caspase-dependent Microtubule Destabilization. J. Neurosci. 38 (2), 363–378. 10.1523/JNEUROSCI.1511-17.2017 29175955 PMC5761614

[B56] Klaiman G. Petzke T. L. Hammond J. LeBlanc A. C. (2008). Targets of Caspase-6 Activity in Human Neurons and Alzheimer Disease. Mol. Cell Proteomics 7 (8), 1541–1555. 10.1074/mcp.M800007-MCP200 18487604 PMC2500235

[B57] Konishi Y. Lehtinen M. Donovan N. Bonni A. (2002). Cdc2 Phosphorylation of BAD Links the Cell Cycle to the Cell Death Machinery. Mol. Cel 9, 1005–1016. 10.1016/s1097-2765(02)00524-5 12049737

[B58] Kuo C. T. Zhu S. Younger S. Jan L. Y. Jan Y. N. (2006). Identification of E2/E3 Ubiquitinating Enzymes and Caspase Activity Regulating Drosophila Sensory Neuron Dendrite Pruning. Neuron 51 (3), 283–290. 10.1016/j.neuron.2006.07.014 16880123

[B59] Kuranaga E. Kanuka H. Tonoki A. Takemoto K. Tomioka T. Kobayashi M. (2006). Drosophila IKK-Related Kinase Regulates Nonapoptotic Function of Caspases via Degradation of IAPs. Cell 126 (3), 583–596. 10.1016/j.cell.2006.05.048 16887178

[B60] Laguna A. Aranda S. Barallobre M. J. Barhoum R. Fernández E. Fotaki V. (2008). The Protein Kinase DYRK1A Regulates Caspase-9-Mediated Apoptosis during Retina Development. Dev. Cel 15 (6), 841–853. 10.1016/j.devcel.2008.10.014 19081073

[B61] Laguna A. Barallobre M. J. Marchena M. Á. Mateus C. Ramírez E. Martínez-Cue C. C. (2013). Triplication of Dyrk1a Causes Retinal Structural and Functional Alterations in Down Syndrome. Hum. Mol. Genet. 22 (14), 2275–2284. 10.1093/hmg/ddt125 23512985

[B62] Li Z. Jo J. Jia J. M. Lo S. C. Whitcomb D. J. Jiao S. (2010). Caspase-3 Activation via Mitochondria Is Required for Long-Term Depression and AMPA Receptor Internalization. Cell 141 (5), 859–871. 10.1016/j.cell.2010.03.053 20510932 PMC2909748

[B63] Liu B. Shi Y. He H. Cai M. Xiao W. Yang X. (2018). miR-221 Modulates Skeletal Muscle Satellite Cells Proliferation and Differentiation. Vitro Cell Dev. Biol. - Anim. 54 (2), 147–155. 10.1007/s11626-017-0210-x 29197032

[B64] Lo S. C. Wang Y. Weber M. Larson J. L. Scearce-Levie K. Sheng M. (2015). Caspase-3 Deficiency Results in Disrupted Synaptic Homeostasis and Impaired Attention Control. J. Neurosci. 35 (5), 2118–2132. 10.1523/JNEUROSCI.3280-14.2015 25653368 PMC6705356

[B65] Lucotte B. Tajhizi M. Alkhatib D. Samuelsson E. B. Wiehager B. Schedin-Weiss S. (2015). Stress Conditions Increase Vimentin Cleavage by Omi/HtrA2 Protease in Human Primary Neurons and Differentiated Neuroblastoma Cells. Mol. Neurobiol. 52 (3), 1077–1092. 10.1007/s12035-014-8906-3 25288153

[B66] Maness P. F. Schachner M. (2007). Neural Recognition Molecules of the Immunoglobulin Superfamily: Signaling Transducers of Axon Guidance and Neuronal Migration. Nat. Neurosci. 10 (1), 19–26. 10.1038/nn1827 17189949

[B67] Maor-Nof M. Romi E. Shalom H. S. Ulisse V. Raanan C. Nof A. (2016). Axonal Degeneration Is Regulated by a Transcriptional Program that Coordinates Expression of Pro- and Anti-degenerative Factors. Neuron 92 (5), 991–1006. 10.1016/j.neuron.2016.10.061 27889097

[B68] Martínez-Mármol R. Barneda-Zahonero B. Soto D. Andrés R. M. Coccia E. Gasull X. (2016). FAIM-L Regulation of XIAP Degradation Modulates Synaptic Long-Term Depression and Axon Degeneration. Scientific Rep. 6. 10.1038/srep35775 PMC5073314 27767058

[B69] Martins L. Iaccarino I. Tenev T. Gschmeissner S. Totty N. F. Lemoine N. R. (2002). The Serine Protease Omi/HtrA2 Regulates Apoptosis by Binding XIAP through a Reaper-like Motif. J. Biol. Chem. 277 (1), 439–444. 10.1074/jbc.M109784200 11602612

[B70] Mashima T. Naito M. Noguchi K. Miller D. K. Nicholson D. W. Tsuruo T. (1997). Actin Cleavage by CPP-32/apopain during the Development of Apoptosis. Oncogene 14, 1001–1012. 10.1038/sj.onc.1200919 9070648

[B71] Miller-Fleming T. W. Petersen S. C. Manning L. Matthewman C. Gornet M. Beers A (2016). The DEG/ENaC cation channel protein UNC-8 drives activity-dependent synapse removal in remodeling GABAergic neurons. *ELife, 5*(e14599). 10.7554/eLife.14599.001 PMC4980115 27403890

[B72] Mori T. Wada T. Suzuki T. Kubota Y. Inagaki N. (2007). Singar1, a Novel RUN Domain-Containing Protein, Suppresses Formation of Surplus Axons for Neuronal Polarity. J. Biol. Chem. 282 (27), 19884–19893. 10.1074/jbc.M700770200 17439943

[B73] Moubarak R. S. Planells-Ferrer L. Urresti J. Reix S. Segura M. F. Carriba P. (2013). FAIM-L Is an IAP-Binding Protein that Inhibits XIAP Ubiquitinylation and Protects from Fas-Induced Apoptosis. J. Neurosci. 33 (49), 19262–19275. 10.1523/JNEUROSCI.2479-13.2013 24305822 PMC6618789

[B74] Mukherjee A. Pop S. Kondo S. Williams D. W. (2021). Proapoptotic RHG Genes and Mitochondria Play a Key Non-apoptotic Role in Remodelling the Drosophila Sensory System. BioRxiv. 10.1101/2021.01.15.426850

[B75] Nikolaev A. McLaughlin T. O’Leary D. D. M. Tessier-Lavigne M. (2009). APP Binds DR6 to Trigger Axon Pruning and Neuron Death via Distinct Caspases. Nature 457 (7232), 981–989. 10.1038/nature07767 19225519 PMC2677572

[B76] Noyan-Ashraf M. H. Brandizzi F. Juurlink B. H. J. (2005). Constitutive Nuclear Localization of Activated Caspase 3 in Subpopulations of the Astroglial Family of Cells. GLIA 49 (4), 588–593. 10.1002/glia.20140 15578657

[B77] Ohsawa S. Hamada S. Asou H. Kuida K. Uchiyama Y. Yoshida H. (2009). Caspase-9 Activation Revealed by Semaphorin 7A Cleavage Is Independent of Apoptosis in the Aged Olfactory Bulb. J. Neurosci. 29 (36), 11385–11392. 10.1523/JNEUROSCI.4780-08.2009 19741144 PMC6665912

[B78] Ohsawa S. Hamada S. Kuida K. Yoshida H. Igaki T. Miura M. (2010). Maturation of the Olfactory Sensory Neurons by Apaf-1/caspase-9-Mediated Caspase Activity. Proc. Natl. Acad. Sci. United States America 107 (30), 13366–13371. 10.1073/pnas.0910488107 PMC2922127 20624980

[B79] Oomman S. Strahlendorf H. Dertien J. Strahlendorf J. (2006). Bergmann Glia Utilize Active Caspase-3 for Differentiation. Brain Res. 1078 (1), 19–34. 10.1016/j.brainres.2006.01.041 16700096

[B80] Öztürk G. Cengiz N. Erdoǧan E. Him A. Oǧuz E. K. Yenidünya E. (2013). Two Distinct Types of Dying Back Axonal Degeneration *In Vitro* . Neuropathol. Appl. Neurobiol. 39 (4), 362–376. 10.1111/j.1365-2990.2012.01295.x 22845867

[B81] Pacheco A. Gallo G. (2016). Actin Filament-Microtubule Interactions in Axon Initiation and Branching. Brain Res. Bull. 126, 300–310. 10.1016/j.brainresbull.2016.07.013 27491623 PMC5518172

[B82] Pakavathkumar P. Noël A. Lecrux C. Tubeleviciute-Aydin A. Hamel E. Ahlfors J. E. (2017). Caspase Vinyl Sulfone Small Molecule Inhibitors Prevent Axonal Degeneration in Human Neurons and Reverse Cognitive Impairment in Caspase-6-Overexpressing Mice. Mol. Neurodegeneration 12 (1), 22. 10.1186/s13024-017-0166-z PMC5329948 28241839

[B83] Pinan-Lucarre B. Gabel C. Reina v. Hulme C. P. Shevkoplyas S. E. Slone S. S. (2012). The Core Apoptotic Executioner Proteins CED-3 and CED-4 Promote Initiation of Neuronal Regeneration in caenorhabditis Elegans. PLoS Biol. 10 (5). 10.1371/journal.pbio.1001331 PMC3358320 22629231

[B84] Pistritto G. Papaleo V. Sanchez P. Ceci C. Barbaccia M. L. (2012). Divergent Modulation of Neuronal Differentiation by Caspase-2 and -9. PLoS ONE 7 (5)). 10.1371/journal.pone.0036002 PMC3356362 22629307

[B85] Potts M. B. Vaughn A. E. McDonough H. Patterson C. Deshmukh M. (2005). Reduced Apaf-1 Levels in Cardiomyocytes Engage Strict Regulation of Apoptosis by Endogenous XIAP. J. Cel Biol. 171 (6), 925–930. 10.1083/jcb.200504082 PMC2171313 16344307

[B86] Ramón Martínez-François J. Fernández-Agü Era C. Nathwani N. Lahmann C. Burnham V. L. Danial N. N. (2018). Bad and K Atp Channels Regulate Neuron Excitability and Epileptiform Activity. ELife 7, e32721. 10.7554/eLife.32721.001 29368690 PMC5785210

[B87] Rebellato P. Kaczynska D. Kanatani S. Rayyes I. Zhang S. Villaescusa C. (2019). The T-type Ca 2+ Channel Ca V 3.2 Regulates Differentiation of Neural Progenitor Cells during Cortical Development via Caspase-3. Neuroscience 402, 78–89. 10.1016/j.neuroscience.2019.01.015 30677486

[B88] Reimertz C. Ko Ègel D. Lankiewicz S. Poppe M. Prehn J. H. M. (2001). Ca2+-induced Inhibition of Apoptosis in Human SH-Sy5y Neuroblastoma Cells: Degradation of Apoptotic Protease Activating Factor-1 (APAF-1). J. Neurochem., 1256–1266. 10.1046/j.1471-4159.2001.00503.x 11579134

[B89] Rossiter J. P. Anderson L. L. Yang F. Cole G. M. Yang F. Cole G. M. (2000). Caspase-cleaved Actin (Fractin) Immunolabelling of Hirano Bodies. Neuropathol. Appl. Neurobiol. 26, 342–346. 10.1046/j.1365-2990.2000.00252.x 10931367

[B90] Rouaux C. Mbebi C. Boutillier S. Loeffler J.-P. Boutillie A.-L. (2003). Critical Loss of CBP/p300 Histone Acetylase Activity by Caspase-6 during Neurodegeneration. EMBO J. 22 (24), 6537–6549. 10.1093/emboj/cdg615 14657026 PMC291810

[B91] Rozenfeld-Granot G. Krishnamurthy J. Kannan K. Toren A. Amariglio N. Givol D. (2002). A Positive Feedback Mechanism in the Transcriptional Activation of Apaf-1 by P53 and the Coactivator Zac-1. Oncogene 21, 1469–1476. 10.1038/sj/onc/120521810.1038/sj.onc.1205218 11896574

[B92] Ruchaud S. (2002). Caspase-6 Gene Disruption Reveals a Requirement for Lamin A Cleavage in Apoptoticchromatin Condensation. EMBO J. 21, 1967–1977. 10.1093/emboj/21.8.1967 11953316 PMC125972

[B93] Schoenmann Z. Assa-Kunik E. Tiomny S. Minis A. Haklai-Topper L. Arama E. (2010). Axonal Degeneration Is Regulated by the Apoptotic Machinery or a NAD +-sensitive Pathway in Insects and Mammals. J. Neurosci. 30 (18), 6375–6386. 10.1523/JNEUROSCI.0922-10.2010 20445064 PMC6632718

[B94] Shan Y. Farmer S. M. Wray S. (2021). Drebrin Regulates Cytoskeleton Dynamics in Migrating Neurons through Interaction with CXCR4. Proc. Natl. Acad. Sci. USA 118 (e2009493118). 10.1073/pnas.2009493118/-/DCSupplemental PMC7826346 33414275

[B95] Shi J. Zhao Y. Wang Y. Gao W. Ding J. Li P. (2014). Inflammatory Caspases Are Innate Immune Receptors for Intracellular LPS. Nature 514 (7521), 187–192. 10.1038/nature13683 25119034

[B96] Shi Y. (2004). Minireview Caspase Activation: Revisiting the Induced Proximity Model. Cell 117, 855–858. 10.1016/j.cell.2004.06.007 15210107

[B97] Shin J. E. Cho Y. Beirowski B. Milbrandt J. Cavalli V. DiAntonio A. (2012). Dual Leucine Zipper Kinase Is Required for Retrograde Injury Signaling and Axonal Regeneration. Neuron 74 (6), 1015–1022. 10.1016/j.neuron.2012.04.028 22726832 PMC3383631

[B98] Shinoda N. Hanawa N. Chihara T. Koto A. Miura M. (2019). Dronc-independent Basal Executioner Caspase Activity Sustains Drosophila Imaginal Tissue Growth. Proc. Natl. Acad. Sci. United States America 116 (41), 20539–20544. 10.1073/pnas.1904647116 PMC6789915 31548372

[B99] Silke J. Meier P. (2013). Inhibitor of Apoptosis(IAP) Proteins-Modulators of Cell Death and Inflammation. Cold Spring Harbor Perspect. Biol. 5 (2). 10.1101/cshperspect.a008730 PMC3552501 23378585

[B100] Simon D. J. Pitts J. Hertz N. T. Yang J. Yamagishi Y. Olsen O. (2016). Axon Degeneration Gated by Retrograde Activation of Somatic Pro-apoptotic Signaling. Cell 164 (5), 1031–1045. 10.1016/j.cell.2016.01.032 26898330 PMC4785881

[B101] Simon D. J. Weimer R. M. Mclaughlin T. Kallop D. Stanger K. Yang J. (2012). A Caspase cascade Regulating Developmental Axon Degeneration. J. Neurosci. 32 (49), 17540–17553. 10.1523/JNEUROSCI.3012-12.2012 23223278 PMC3532512

[B102] Sinha S. Levine B. (2008). The Autophagy Effector Beclin 1: A Novel BH3-Only Protein. Oncogene 27, S137–S148. 10.1038/onc.2009.51 19641499 PMC2731580

[B103] Slee E. A. Adrain C. Martin S. J. (1999). Serial Killers: Ordering Caspase Activation Events in Apoptosis. Cel Death Differ. 6, 1067–1074. 10.1038/sj.cdd.4400601 10578175

[B104] Smith M. I. Huang Y. Y. Deshmukh M. (2009). Skeletal Muscle Differentiation Evokes Endogenous XIAP to Restrict the Apoptotic Pathway. PLoS ONE 4 (3). 10.1371/journal.pone.0005097 PMC2658743 19333375

[B105] Stepanichev M. Y. Kudryashova I. Yakovlev A. A. Onufriev M. v. Khaspekov L. G. Lyzhin A. A. (2005). Central Administration of a Caspase Inhibitor Impairs Shuttle-Box Performance in Rats. Neuroscience 136 (2), 579–591. 10.1016/j.neuroscience.2005.08.010 16198488

[B106] Strauss K. M. Martins L. M. Plun-Favreau H. Marx F. P. Kautzmann S. Berg D. (2005). Loss of Function Mutations in the Gene Encoding Omi/HtrA2 in Parkinson’s Disease. Hum. Mol. Genet. 14 (15), 2099–2111. 10.1093/hmg/ddi215 15961413

[B107] Uguagliati B. Al-Absi A. R. Stagni F. Emili M. Giacomini A. Guidi S. (2021). Early Appearance of Developmental Alterations in the Dendritic Tree of the Hippocampal Granule Cells in the Ts65Dn Model of Down Syndrome. Hippocampus 31 (4), 435–447. 10.1002/hipo.23303 33464704

[B108] Unsain N. Higgins J. M. Parker K. N. Johnstone A. D. Barker P. A. (2013). XIAP Regulates Caspase Activity in Degenerating Axons. Cel Rep. 4 (4), 751–763. 10.1016/j.celrep.2013.07.015 23954782

[B109] Uribe V. Wong B. K. Y. Graham R. K. Cusack C. L. Skotte N. H. Pouladi M. A. (2012). Rescue from Excitotoxicity and Axonal Degeneration Accompanied by Age-dependent Behavioral and Neuroanatomical Alterations in Caspase-6-Deficient Mice. Hum. Mol. Genet. 21 (9), 1954–1967. 10.1093/hmg/dds005 22262731 PMC3315204

[B110] Varghese J. Radhika G. Sarin A. (2001). The Role of Calpain in Caspase Activation during Etoposide Induced Apoptosis in T Cells. Eur. J. Immunol. 31, 2035–2041. 10.1002/1521-4141(200107)31:7<2035::aid-immu2035>3.0.co;2-y 11449356

[B111] Verma P. Chierzi S. Codd A. M. Campbell D. S. Meyer R. L. Holt C. E. (2005). Axonal Protein Synthesis and Degradation Are Necessary for Efficient Growth Cone Regeneration. J. Neurosci. 25 (2), 331–342. 10.1523/JNEUROSCI.3073-04.2005 15647476 PMC3687202

[B112] Victor K. G. Heffron D. S. Sokolowski J. D. Majumder U. Leblanc A. Mandell J. W. (2018). Proteomic Identification of Synaptic Caspase Substrates. Synapse 72 (1), e22014. 10.1002/syn.22014 28960461

[B113] Virdee K. Parone P. A. Tolkovsky A. M. (2000). Phosphorylation of the Pro-apoptotic Protein BAD on Serine 155, a Novel Site, Contributes to Cell Survival. Curr. Biol. 10, 1151–1154. 10.1016/s0960-9822(00)00702-8 10996800

[B114] Vohra B. P. S. Sasaki Y. Miller B. R. Chang J. DiAntonio A. Milbrandt J. (2010). Amyloid Precursor Protein Cleavage-dependent and -independent Axonal Degeneration Programs Share a Common Nicotinamide Mononucleotide Adenylyltransferase 1-sensitive Pathway. J. Neurosci. 30 (41), 13729–13738. 10.1523/JNEUROSCI.2939-10.2010 20943913 PMC3104322

[B115] Walsh F. S. Doherty P. (1997). Neural Cell Adhesion Molecules of the Immunoglobulin Superfamily: Role in Axon Growth and Guidance. Annu. Rev. Cel Dev. Biol 13, 425–456. 10.1146/annurev.cellbio.13.1.425 9442880

[B116] Wang G. Zhang Q. Song Y. Wang X. Guo Q. Zhang J. (2015). PAK1 Regulates RUFY3-Mediated Gastric Cancer Cell Migration and Invasion. Cel Death Dis. 6, e1682. 10.1038/cddis.2015.50 PMC4385928 25766321

[B117] Wang X. J. Cao Q. Liu X. Wang K. T. Mi W. Zhang Y. (2010). Crystal Structures of Human Caspase 6 Reveal a New Mechanism for Intramolecular Cleavage Self-Activation. EMBO Rep. 11 (11), 841–847. 10.1038/embor.2010.141 20890311 PMC2966951

[B118] Wang Y. J. Liu M. G. Wang J. H. Cao W. Wu C. Wang Z. Y. (2020). Restoration of Cingulate Long-Term Depression by Enhancing Non-apoptotic Caspase 3 Alleviates Peripheral Pain Hypersensitivity. Cel Rep. 33 (6). 10.1016/j.celrep.2020.108369 33176141

[B119] Weaver B. P. Zabinsky R. Weaver Y. M. Lee E. S. Xue D. Han M. (2014). CED-3 Caspase Acts with miRNAs to Regulate Non-apoptotic Gene Expression Dynamics for Robust Development in *C. elegans* . ELife 3 (November), 1–22. 10.7554/eLife.04265 PMC4279084 25432023

[B120] Weghorst F. Mirzakhanyan Y. Samimi K. Dhillon M. Barzik M. Cunningham L. L. (2020). Caspase-3 Cleaves Extracellular Vesicle Proteins during Auditory Brainstem Development. Front. Cell Neurosci. 14. 10.3389/fncel.2020.573345 PMC7689216 33281555

[B121] Wei Z. Sun M. Liu X. Zhang J. Jin Y. (2014). Rufy3, a Protein Specifically Expressed in Neurons, Interacts with Actin-Bundling Protein Fascin to Control the Growth of Axons. J. Neurochem. 130 (5), 678–692. 10.1111/jnc.1258010.1111/jnc.12740 24720729

[B122] Westphal D. Sytnyk V. Schachner M. Leshchyns’ka I. (2010). Clustering of the Neural Cell Adhesion Molecule (NCAM) at the Neuronal Cell Surface Induces Caspase-8- and -3-dependent Changes of the Spectrin Meshwork Required for NCAM-Mediated Neurite Outgrowth. J. Biol. Chem. 285 (53), 42046–42057. 10.1074/jbc.M110.177147 20961848 PMC3009930

[B123] Williams D. W. Kondo S. Krzyzanowska A. Hiromi Y. Truman J. W. (2006). Local Caspase Activity Directs Engulfment of Dendrites during Pruning. Nat. Neurosci. 9 (10), 1234–1236. 10.1038/nn1774 16980964

[B124] Wong B. K. Y. Ehrnhoefer D. E. Graham R. K. Martin D. D. O. Ladha S. Uribe V. (2015). Partial rescue of Some Features of Huntington Disease in the Genetic Absence of Caspase-6 in YAC128 Mice. Neurobiol. Dis. 76, 24–36. 10.1016/j.nbd.2014.12.030 25583186

[B125] Wright K. M. Linhoff M. W. Potts P. R. Deshmukh M. (2004). Decreased Apoptosome Activity with Neuronal Differentiation Sets the Threshold for Strict IAP Regulation of Apoptosis. J. Cel Biol. 167 (2), 303–313. 10.1083/jcb.200406073 PMC2172554 15504912

[B126] Yan D. Wu Z. Chisholm A. D. Jin Y. (2009). The DLK-1 Kinase Promotes mRNA Stability and Local Translation in *C. elegans* Synapses and Axon Regeneration. Cell 138 (5), 1005–1018. 10.1016/j.cell.2009.06.023 19737525 PMC2772821

[B127] Yin V. P. Thummel C. S. Bashirullah A. (2007). Down-regulation of Inhibitor of Apoptosis Levels Provides Competence for Steroid-Triggered Cell Death. J. Cel Biol. 178 (1), 85–92. 10.1083/jcb.200703206 PMC2064425 17591924

[B128] Yuan J. (1993). The *C. elegans* Cell Death Gene Ced-3 Encodes a Protein Similar to Mammalian Interleukin-1β-Converting Enzyme. Cell 75 (4), 641–652. 10.1016/0092-8674(93)90485-9 8242740

[B129] Ziv N. E. Spira M. E. (1995). Axotomy Induces a Transient and Localized Elevation of the Free Intracellular Calcium Concentration to the Millimolar Range. J. Neurophysiol. 74 (6), 2625–2637. 10.1152/jn.1995.74.6.2625 8747220

